# Forensic electrochemistry on a drop: bromocresol green-modified screen-printed platforms for dual colorimetric-electrochemical detection of scopolamine

**DOI:** 10.1039/d6ra07984a

**Published:** 2026-10-06

**Authors:** Larissa M. A. Melo, Robert D. Crapnell, Elena Bernalte, Daniel S. Paula, Rodrigo A. A. Munoz, Wendell K. T. Coltro, Wallans T. P. dos Santos, Craig E. Banks

**Affiliations:** a Faculty of Science and Engineering, Manchester Metropolitan University Dalton Building, Chester Street M1 5GD UK c.banks@mmu.ac.uk; b Department of Chemistry, Federal University of Vales Do Jequitinhonha and Mucuri Campus JK 39100-000 Diamantina Minas Gerais Brazil; c Institute of Chemistry, Federal University of Goiás Campus Samambaia 74690-900 Goiânia Goiás Brazil; d Institute of Chemistry, Federal University of Uberlândia 38400-902 Uberlândia Minas Gerais Brazil; e Department of Pharmacy, Federal University of Vales Do Jequitinhonha and Mucuri Campus JK 39100000 Diamantina Minas Gerais Brazil wallanst@ufvjm.edu.br

## Abstract

A novel bulk-modified screen-printed electrode (SPE) was developed by incorporating bromocresol green (BG) as a pH-responsive colorimetric reagent, directly into the graphite ink formulation to create an integrated dual-response sensing platform. This bulk modification strategy enables complementary electrochemical and colorimetric responses on a single disposable device (BG-SPE), eliminating the need for additional reagents or multi-step procedures. The platform was applied to the detection of scopolamine, a clinically relevant tropane alkaloid associated with both therapeutic use and drug-facilitated criminal activity. The BG-SPE sensor enabled rapid, portable preliminary visual screening followed by electrochemical confirmation and quantification of scopolamine in beverages and biological matrices. Differential pulse voltammetry provided a wide linear range from 5 to 80 µg mL^−1^, with limits of detection (3*σ*) and quantification (10*σ*) of 0.02 and 0.07 µg mL^−1^, respectively, alongside excellent repeatability (RSD < 7.0% for peak current and <2.0% for peak potential). Method validation in real beverage samples (whiskey) and synthetic biological fluids (saliva, urine, and vitreous humour) yielded recoveries close to 100% with negligible matrix effects. The bulk incorporation of BG into the ink formulation provided reproducible electrode fabrication, supporting the use of bulk-modified SPE for on-site forensic screening, while allowing both analytical responses to be generated from the same sensing interface. The bulk-modified SPE integrates visual screening and electrochemical confirmation and quantification within a single portable sensing platform, enabling complementary analytical information to be obtained from the same sensing interface without requiring addition reagents.

## Abstract 2

A novel bulk-modified screen-printed electrode (SPE) was developed by incorporating bromocresol green (BG), as a pH-responsive colorimetric reagent, directly into the graphite ink formulation to create an integrated dual-response sensing platform.

## Graphical abstract

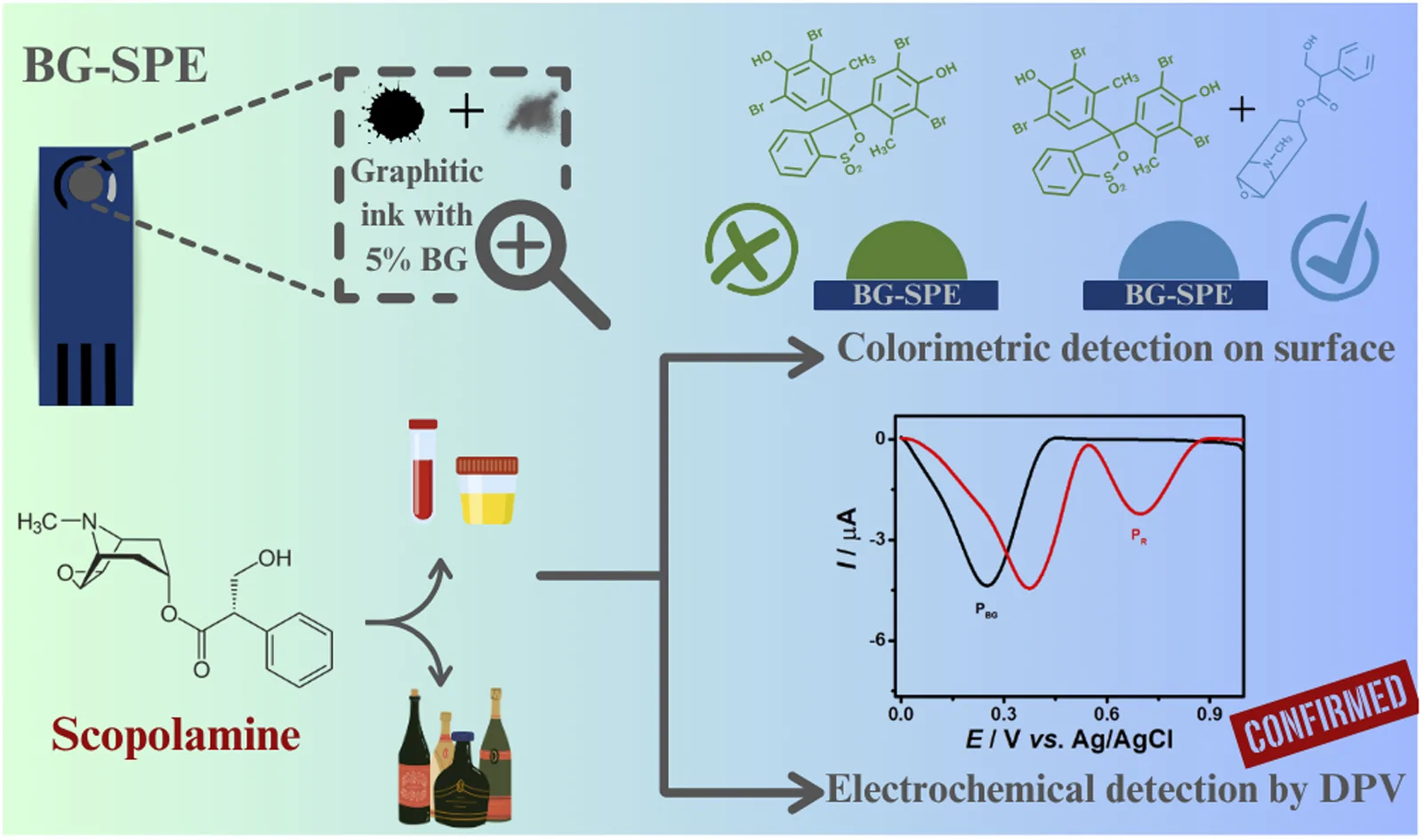

## Introduction

Drug-facilitated sexual assault involves the use of substances that induce amnesia, profound sedation, or otherwise impair a victim's ability to resist or recall events. Among these agents, scopolamine is particularly notorious in forensic toxicology, as its strong anticholinergic effects cause confusion, memory loss, and reduced motor control, thereby explaining its frequent implication in criminal contexts, especially in cases of drug-facilitated robbery and sexual assault.^1,2^ Scopolamine has emerged as a substance of concern due to its rapid hepatic metabolism, short plasma half-life, and instability at room temperature, all of which contribute to a narrow detection window in forensic samples.^3^ The United Nations Office on Drugs and Crime (UNODC) recommends liquid chromatography coupled to mass spectrometry (LC-MS) and gas chromatography coupled to mass spectrometry (GC-MS) as reference techniques for scopolamine detection, given their high selectivity in forensic toxicology.^4^ These methods involve lengthy processing and validation protocols, which may compromise analytical reliability if biological samples are not collected promptly and stored under appropriate conditions.^5,6^ In this regard, on-site analytical approaches are particularly relevant in forensic contexts, where sample collection, transportation, preparation, and subsequent laboratory analysis are often impractical, and rapid *in situ* detection can provide critical support for timely decision-making.

Given these limitations, rapid and selective screening methods represent valuable preliminary tools for suspected scopolamine poisoning, offering portability and immediate analytical results. Electrochemical techniques are particularly appropriate in this context owing to their intrinsic sensitivity to redox-active species, minimal sample preparation requirements, and potential for integration into portable platforms. Electrochemical methods for scopolamine detection have employed boron-doped diamond electrodes,^7,8^ carbon-paste electrodes modified with mesostructured silica,^9^ and platinum electrodes.^10^ These conventional electrodes present practical challenges for *in situ* applications, including the necessity for frequent polishing and cleaning, which may compromise reproducibility and operational efficiency. By contrast, screen-printed electrodes (SPEs) offer disposability, thereby minimizing the risk of contamination and false positives, while providing simplicity, versatility, and ease of integration into portable analytical systems.^11,12^

Furthermore, recent advances have investigated the integration of colorimetric and electrochemical techniques, combining the visual simplicity of colour-based assays with the high sensitivity and selectivity afforded by voltammetric detection.^13,14^ This dual-modality approach enhances the reliability of analytical outcomes by merging responses from two distinct yet complementary techniques. Such strategies have already been applied to analytes including 3,4-methylenedioxymethamphetamine (MDMA), methamphetamine, lysergic acid diethylamide, phenylethylamine-type drugs, atropine, and scopolamine itself using reagents such as Dragendorff's.^15–18^ Although a previous study demonstrated the feasibility of combining independent colorimetric and electrochemical measurements for scopolamine screening,^17^ the colorimetric step relied on the external addition of Dragendorff's reagent and was therefore conducted independently of the electrochemical measurement. More generally, dual-response methodologies reported to date typically require separate analytical procedures, often performed on different platforms, resulting in additional handling steps and preventing true integration of both detection modes. Consequently, there remains a need for a sensing platform in which the colorimetric functionality is incorporated directly into the electrode architecture, enabling both visual and electrochemical information to be generated from a single disposable device without the addition of external reagents.

In this context, bromocresol green (BG) is an organic dye widely used across analytical chemistry, biochemistry, and environmental sciences and it is commonly used as a pH indicator, in protein quantification, selective substance identification, and water quality analysis. Beyond these conventional applications, BG has also been reported as a colorimetric reagent for atropine detection^18^ and as a modification material for working electrodes in electrochemical sensors through electropolymerized polymer films.^19^ In electroanalytical systems, BG can enhance selectivity and promote charge-transfer processes, thereby facilitating the detection of target analytes, including drugs and environmental contaminants. Electropolymerized poly(bromocresol green) films have been successfully applied for the sensing of a range of analytes,^2,19–22^ highlighting the suitability of BG for integrated colorimetric-electrochemical sensing platforms. However, despite the widespread use of electropolymerization for electrode modification, practical limitations associated with film thickness control, reproducibility, and long-term stability continue to restrict broader implementation.^23,24^

The drop-casting technique is widely reported for electrode modification in electrochemical sensors due to its simplicity and cost-effectiveness. However, this method presents several limitations, including poor reproducibility, uneven surface coverage, and low mechanical stability, which can compromise the reliability and performance of the sensors.^25^ In contrast, ink incorporation techniques, such as inkjet printing and screen-printing, offer distinct advantages. These methods enable precise control over film thickness and uniformity, enhancing the mechanical stability and scalability of the sensors. Furthermore, ink incorporation is particularly suited for mass production, facilitating the development of cost-effective and reliable electrochemical sensors.^26^

To address the demand for integrated analytical platforms, we report, for the first time, a bulk modified screen-printed electrode fabricated by directly incorporating BG into the graphite ink, thereby integrating the colorimetric reagent into the sensor architecture. Unlike conventional dual-response approaches, where the sensing surface and the colorimetric reagent are physically separated, the present strategy transforms the working electrode itself to the reactive colorimetric interface. To demonstrate the applicability of the proposed sensing concept, the bulk modified BG-SPE platform was applied to the detection of scopolamine in beverages and biological samples. To the best of our knowledge, this is the first study to incorporate BG directly into graphite ink, enabling simultaneous colorimetric and electrochemical detection within a single platform. This design introduces a different sensing concept, in which the colorimetric reagent becomes an integral component of the electrode rather than an externally added analytical reagent.

## Materials and methods

All reagents were of analytical standard (purity ≥98%) obtained from Sigma-Aldrich (Lancashire, UK). A stock solution of 1.0 mg mL^−1^ (3.3 mmol L^−1^) scopolamine was prepared by dissolving the compound in methanol, followed by a 10-fold dilution in a supporting electrolyte for subsequent electrochemical analysis. Electrochemical investigations were performed in a 0.1 mol L^−1^ Britton–Robinson (BR) buffer, an equimolar mixture of boric, phosphoric, and acetic acids, with the pH adjusted between 2.0 and 12.0 using 1.0 mol L^−1^ sodium hydroxide. Ammonium chloride (10 mmol L^−1^) was used as supporting electrolyte. A redox probe of 1.0 mmol L^−1^ [Fe(CN)_6_]^4−^/0.1 mol L^−1^ KCl was used to calculate the electroactive area. All solutions were prepared using deionized water with a resistivity of not less than 18.2 MΩ cm^−1^, sourced from a Milli-Q Integral 3 system (Millipore, Watford, UK).

Ascorbic acid (AA), citric acid (CA), uric acid (UA), glucose (GLU), fructose (FRU), paracetamol (PAR), caffeine (CAF), and creatine were evaluated by the proposed method as possible interferents. All reagents were of analytical grade and were acquired from Sigma-Aldrich (Lancashire, UK). Additionally, drinks (whiskeys) and synthetic biological samples, such as artificial saliva, synthetic urine, and artificial vitreous humour, were prepared according to protocols described in the literature and spiked with 50 µg mL^−1^ of scopolamine. Surface characterization techniques and electrochemical measurements are further detailed in the SI.

## Design and fabrication of the bulk modified bromocresol green screen-printed platforms

The fabrication strategy involved bulk modification, in which the colorimetric reagent was incorporated into the graphite ink prior to printing rather than deposited onto the electrode surface after fabrication. Screen-printed electrodes (SPEs) were fabricated in-lab using custom stencil designs to create a working electrode with a diameter of 3.1 mm. Graphite ink (Product Code: C2000802P2; Gwent Electronic Materials Ltd, UK) was printed onto an ultra-flexible polyester film (Autostat AM flexible polyester substrate 150 µm thickness) using a microDEK 1760RS screen-printing machine (DEK, Weymouth, UK) (step 1). The printed layer was cured in a ventilated oven at 60 °C for 1 hour. Subsequently, Ag/AgCl ink (product code: C2030812P3; Gwent Electronic Materials Ltd, UK) was applied as a pseudo-reference electrode (step 2), followed by a second curing step under the same conditions. To insulate the connections, a dielectric paste (Product Code: D2070423D5; Gwent Electronic Materials Ltd, UK) was printed onto the polyester substrate (step 3), and the SPEs underwent a third curing process under the same conditions. Finally, the working electrode was modified with a mixture of graphite ink and 5 wt% bromocresol green (Tokyo Chemical Industry – Oxford, UK) (step 4). Lower and higher bromocresol green loadings were also evaluated during ink preparation, and 5 wt% was selected because it provided the desired colorimetric response while maintaining suitable printability under the conditions employed. A final curing step at 60 °C for 1 hour was performed, after which the bulk modified BG-SPE electrodes were ready for use. The fabrication steps of bulk modified BG-SPEs are summarised in Fig. 1.

**Fig. 1:**
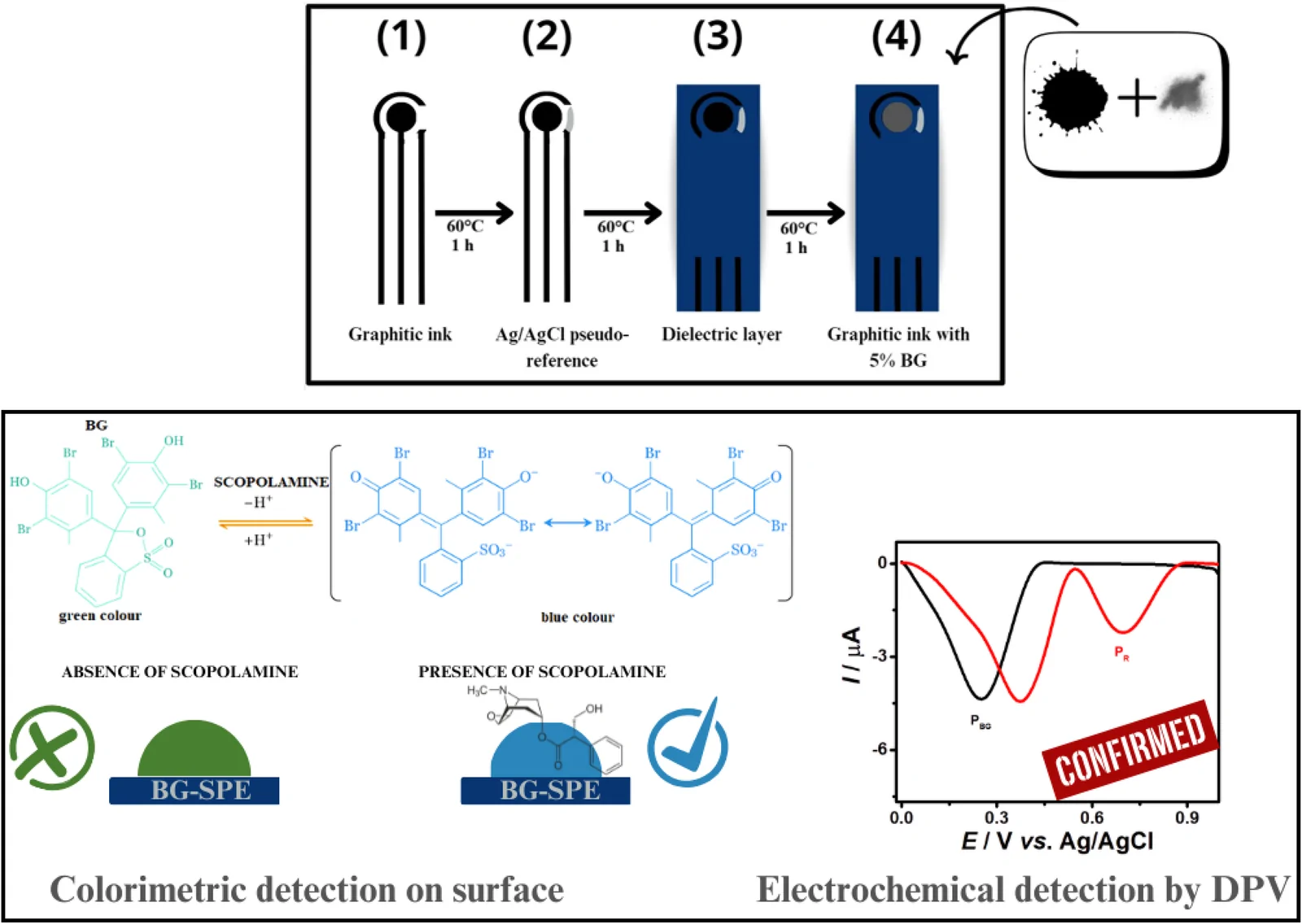
Illustration showing an overview of the fabrication steps required to produce the bulk modified BG-SPE and the scopolamine detection.

## Results and discussion

### Characterization of the bulk modified bromocresol green screen-printed electrodes (BG-SPEs)

The bulk-modified bromocresol green screen-printed electrodes are described in detail in the Experimental Section. Briefly, bromocresol green was incorporated directly into the graphite screen-printing ink at 5 wt%, after which the modified ink was screen-printed and cured to produce the final electrochemical platform. A comprehensive physicochemical characterization of the screen-printed graphite electrodes, both before and after modification with bromocresol green, was conducted using EDX, XPS, Raman spectroscopy, and SEM, as shown in Fig. 2. Raman spectroscopy was used to characterize the SPE and bulk modified BG-SPEs, with the corresponding spectra presented in Fig. 2A. The graphite electrodes, both unmodified and modified, exhibited characteristic Raman bands at approximately D (∼1353 cm^−1^), G (∼1580 cm^−1^), and 2D (∼2717 cm^−1^), which are typical of graphite-like structures. The *I*_D_/*I*_G_ ratios were 0.81 for SPE and 0.76 for bulk modified BG-SPEs, indicating a moderate degree of structural disorder in the graphitic materials, as evidenced by the pronounced intensity of the D band. SEM images of the SPE electrodes before modification (Fig. 2B and S1A) and after modification with BG (Fig. 2C and S1B) showed differences in surface morphology, although the heterogeneous nature of screen-printed graphite surfaces prevents definitive conclusion regarding an overall increase in defect density based solely on these images.

**Fig. 2:**
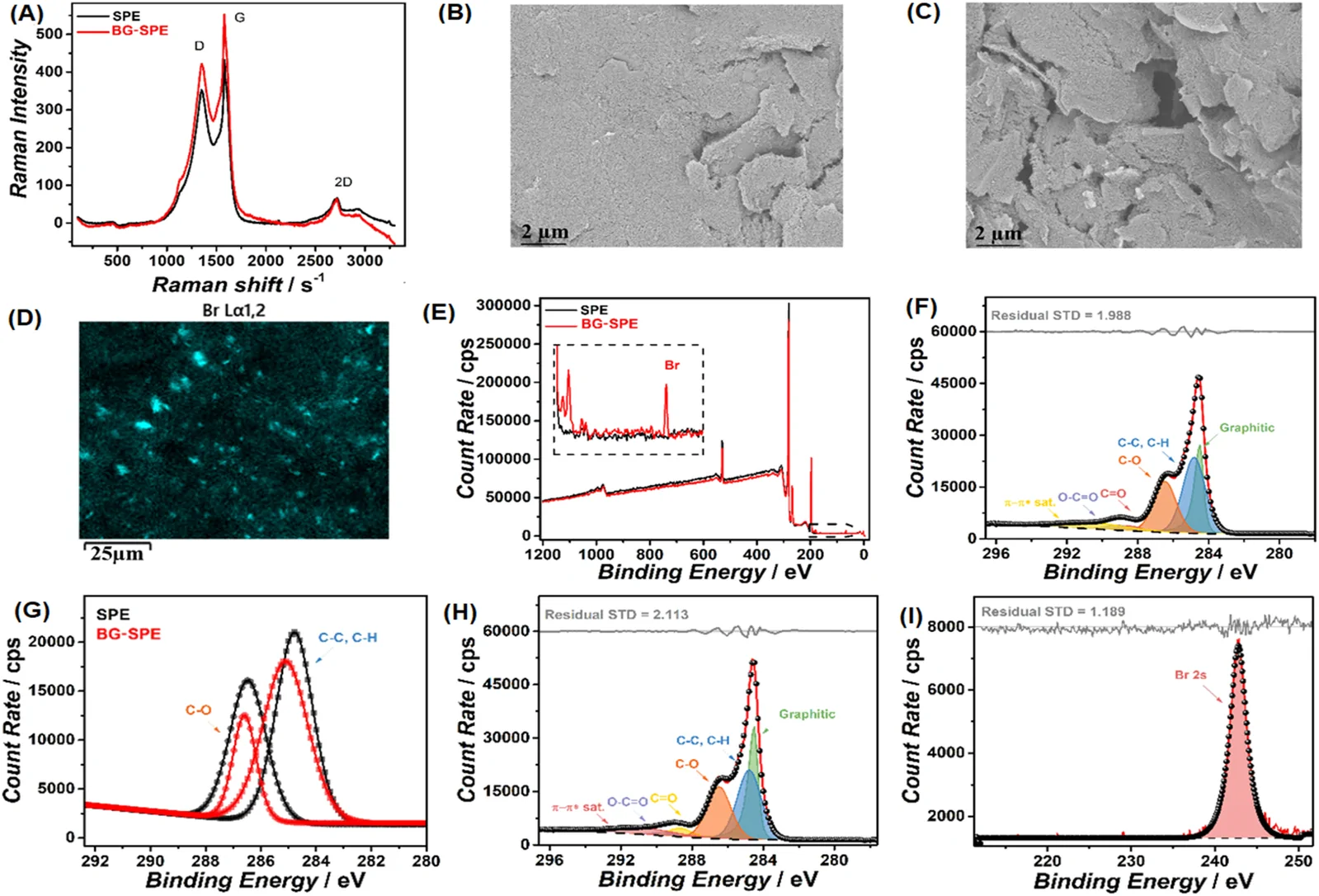
(A) Raman spectra of SPE (black) and bulk modified BG-SPEs (red); SEM imagine of the (B) SPE working electrode surface and (C) of the bulk modified BG-SPE working electrode surface (both at 5.00 k× magnification); (D) SEM-EDX elemental mapping/analysis confirming bromine (Br) presence in bulk modified BG-SPE; (E) XPS survey spectrum of bulk modified BG-SPE; and high-resolution XPS C 1s spectrum of bulk modified BG-SPE (F), for SPE (black) and bulk modified BG-SPE (red) (G), for SPE only (H), and Br 2 s spectra for the bulk modified BG-SPE (I).

The EDX analysis shown in Fig. 2D confirms the successful incorporation of bromocresol green into the bulk modified BG-SPEs through the detection of bromine on the electrode surface. Carbon remained the major constituent of both electrodes, decreasing slightly from 96.5 wt% in the SPE to 90.9 wt% in the BG-SPE. The findings corroborate the successful incorporation of BG into graphite ink. Consistent with the EDX results, XPS analysis of the SPE electrodes (Fig. 2F–I) was consistent with previously reported C 1s spectra.^27^ The primary peak at approximately 284.5 eV corresponds to sp^2^-bonded graphitic C=C structures, while the peak at ∼284.78 eV is associated with edge-plane-like sites or defects related to C–C and C–H bonds. Addition peaks corresponding to C–O, C=O, O–C=O and 
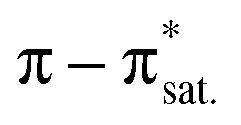
 transitions are commonly observed in graphite-based SPEs. The π–π* contribution arises from the graphitic carbon itself, whereas the oxygen-containing groups originate from the polymeric binders employed in SPE fabrication. This layer is typically composed of various hydrocarbons with minor oxygen functionalities.^28,29^ A slight shift in the C 1s peaks was observed for bulk modified BG-SPEs compared to the unmodified electrode (Fig. 2G), suggesting a possible charge transfer interaction between C and Br.^30^ To further confirm the modification of SPE with BG, XPS analysis for SPE and bulk modified BG-SPEs and for Br 2s is shown in Fig. 2E and I, where the presence of bromine is evident. These results confirm that bromocresol green was successfully incorporated onto the working electrode surface.

Following physicochemical characterization, the electrochemical performance of the electrodes was assessed. This was carried out through scan rate studies using the inner-sphere redox probe [Fe(CN)_6_]^4−^ (1.0 mmol L^−1^/0.1 mol L^−1^ KCl), enabling estimation of the heterogeneous electron transfer rate constant (*k*^0^) and the effective electrochemical surface area (*A*_e_). Representative scan rate studies (5–250 mV s^−1^) for SPE and bulk modified BG-SPEs are presented in Fig. S2A and B, respectively, with scans at 5 mV s^−1^ shown in Fig. S2C. The comparison shows a slight improvement in peak currents and peak-to-peak separations (Δ*E*_p_) for bulk modified BG-SPEs relative to SPE. At 5 mV s^−1^, the measured Δ*E*_p_ bulk modified BG-SPEs is 119 ± 3 mV, compared to 139 ± 4 mV for SPE. A small increase in the electroactive surface area was also observed, from 0.06 (±0.0026) cm^2^ for SPE to 0.07 (±0.0003) cm^2^ for bulk modified BG-SPEs. Although no significant increase in electroactive area was observed, the primary purpose of the BG modification is to provide a colorimetric response alongside the electrochemical signal, rather than solely enhancing electrochemical performance. Consequently, the bulk-modified BG-SPEs exhibit electrochemical behavior comparable to unmodified SPEs, while additionally enabling a colorimetric response through the bromocresol green reaction at the working electrode surface.

### Application of the bulk modified bromocresol green screen-printed electrodes (BG-SPEs) for scopolamine detection: electrochemical and colorimetric behavior of scopolamine

Cyclic voltammetry was employed to investigate the electrochemical behavior of scopolamine across a pH range of 2.0 to 12.0, as shown in Fig. S3A. Note that the bulk modified BG-SPEs itself exhibits certain redox processes characteristic of BG (*P*_BG_). However, in the presence of scopolamine, the oxidation and reduction processes are observed. A change in behavior occurred at pH 6.0, where the reduction process (*P*_R_) becomes difficult to detect and the oxidation process (*P*_O_) was observed only at pH values above 6.0. This behavior is consistent with scopolamine p*K*_a_ ∼ 6.45.^31^ A pH-dependent behavior was observed, with peak potential (*E*_p_) shifting to more negative values as pH increased, as illustrated in Fig. S3B, with the linear regression equations: *E*_p_ (V) = 1.16 (±0.05) − 0.022 (±0.005) × pH for *P*_O_ (pH range 8.0–12.0, *R*^2^ = 0.94) and *E*_p_ (V) = 0.91 (±0.06) − 0.049 (±0.017) × pH for *P*_R_ (pH range 2.0–5.0, *R*^2^ = 0.90). Ammonium chloride was selected as the supporting electrolyte because the BG-based colorimetric response depends on scopolamine-induced changes in solution pH. Consequently, buffered media were avoided, as they could interfere with the pH-dependent colorimetric process. A 10 mmol L^−1^ ammonium chloride solution provided well-defined voltammetric peaks and sufficient peak current, while preserving the visible colour change associated with the BG response arising from the interaction between scopolamine and the sensing interface (Fig. 3). The immediate colour change upon applying the solution to the BG-SPE surface provides a preliminary response, while the voltammetric scan confirms the presence of scopolamine in the solution. Under these conditions, both the colorimetric response and the electrochemical behavior of scopolamine could be simultaneously monitored using the bulk-modified BG-SPE platform.

**Fig. 3:**
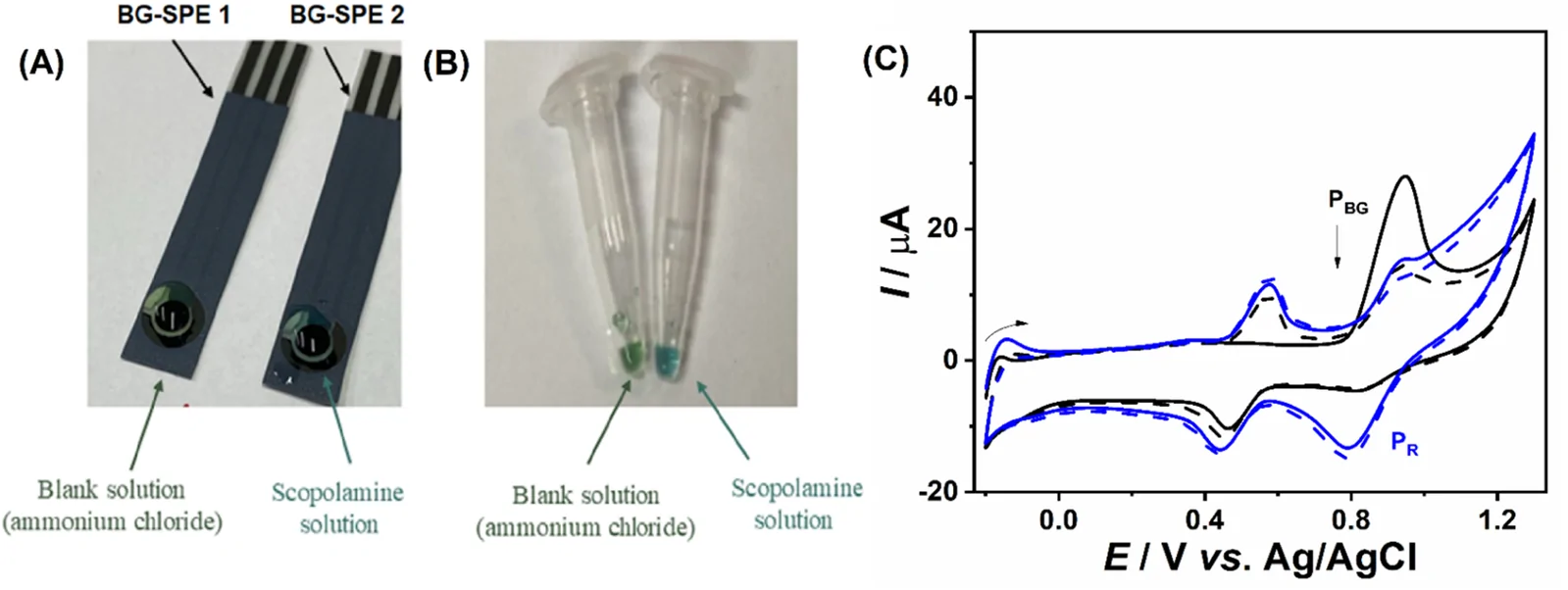
Results for colorimetric reaction with 0.01 mol L^−1^ ammonium chloride solution before (green) and after addition of 0.1 mg mL^−1^ scopolamine (blue) on bulk modified BG-SPEs (A) and in Eppendorf (B). (C) Cyclic voltammograms for 0.01 mol L^−1^ ammonium chloride solution in the absence (black) and in the presence of 0.1 mg mL^−1^ scopolamine (blue) on a bulk BG-SPE; solid lines first scan and dashed lines second scan. All potential scans started at −0.2 V, with a scan rate of 25 mV s^−1^.

It is worth noting that the dark blue insulating ink used on the electrode may hinder visualisation of the BG colour change; therefore, the solutions were transferred to Eppendorf tubes to ensure adequate image acquisition. Alternative insulating ink colours, particularly white, could improve visual contrast and facilitate observation of the BG colour change. Furthermore, the electrode area could potentially be defined using transparent or white insulating materials, such as nail polish, which would be expected to improve visualisation while maintaining the electrode architecture and electrochemical performance.

As shown in Fig. 3A and B, exposure of the BG-modified electrode to scopolamine produced a distinct colour transition from green to blue. This behavior is consistent with the pH-dependent acid-base equilibrium of BG and suggests that scopolamine increases the local pH of the ammonium chloride medium (Scheme S1), thereby inducing the observed colour change. The presence of scopolamine also altered the electrochemical behavior of the BG redox processes (Fig. 3C). Reducing the intensity of the *P*_BG_ peak current response by 55% and the onset of *P*_R_ at +0.79 V (*vs.* Ag/AgCl).

The redox potentials of the BG are pH-dependent, and their behavior changes near the p*K*_a_ of BG (6.57), reflecting the involvement of both protons and electrons in the redox processes.^18^ The shift in the BG redox potential (Fig. 3C) in the presence of scopolamine is likely associated with changes in the local acid-base environment induced by scopolamine, which may alter the protonation state and, consequently, the electrochemical behavior of BG. In a previous study, NMR analysis demonstrated structural changes in BG in the presence of atropine,^18^ including changes consistent with a mesomeric effect. These findings provide a basis for interpreting the similar behavior observed with scopolamine, suggesting that changes in the protonation state and electronic structure of BG may contribute to the observed shift in redox potential.

Unlike previously reported studies, which require the independent addition of a colorimetric reagent in a separate step, the proposed bulk-modified BG-SPE incorporates the colorimetric reagent directly into the electrode architecture. Consequently, the working electrode acts simultaneously as the reaction interface, the colorimetric reagent, and the electrochemical transducer, enabling the acquisition of complementary optical and electrochemical information from a single disposable platform.

It is worth noting that, in the presence of scopolamine, not only does the *P*_R_ reduction process occur, but the BG electrochemical processes also exhibit significant changes. Specifically, the redox couple undergoes a noticeable potential shift (*E*_pBG_), and the oxidation peak shows a significant decrease in current. These differences occur only in the presence of scopolamine and can serve as an additional qualitative indicator of its presence. Therefore, the *P*_R_ peak was selected for the identification and quantification of scopolamine, whereas the potential shift of the BG redox pair provides an additional analytical response to confirm the reaction between scopolamine and BG during the electrochemical step. Next, to investigate the mass-transport behavior of the scopolamine electrochemical process under the given conditions, cyclic voltammetry was performed at scan rates (*v*) ranging from 10 to 600 mV s^−1^, as shown in Fig. S4A. The peak current (*I*_p_) for *P*_R_ exhibited a stronger correlation with the square root of the scan rate (*R*^2^ = 0.99, Fig. S4C) compared to the scan rate itself (*R*^2^ = 0.95, Fig. S4B), indicating that the electrochemical process is predominantly diffusion-controlled at the bulk modified BG-SPE surface. Additionally, the logarithmic plot of *I*_p_*versus v* displayed a linear relationship (Fig. S4D), with linear regression equation: log *I*_p_ (µA) = −5.19 (±0.03) + 0.38 (±0.01) × log *v* (mV s^−1^) and *R*^2^ = 0.986; the slope (≤0.5) further confirms that the reduction process is primarily diffusion-controlled.

It is important to note that some deviations were observed at lower scan rates, particularly in Fig. S4B (*I*_p_*vs. v*). These deviations do not alter the overall interpretation of predominantly diffusion-controlled behavior but suggest that additional non-ideal contributions may become relevant at low scan rates.

### Scopolamine detection by differential pulse voltammetry

Repeatability and reproducibility tests were conducted using the same (*N* = 3) and different (*N* = 5) bulk modified BG-SPEs under optimal conditions (90 mV amplitude, 10 mV step potential, 50 ms modulation time and 0.2 s time interval). Fig. 4 presents the voltammetric peaks obtained by differential pulse voltammetry for *P*_R_ (+0.70 V *vs.* Ag/AgCl), along with the relative standard deviations (RSD) for *I*_p_ and *E*_p_.

**Fig. 4:**
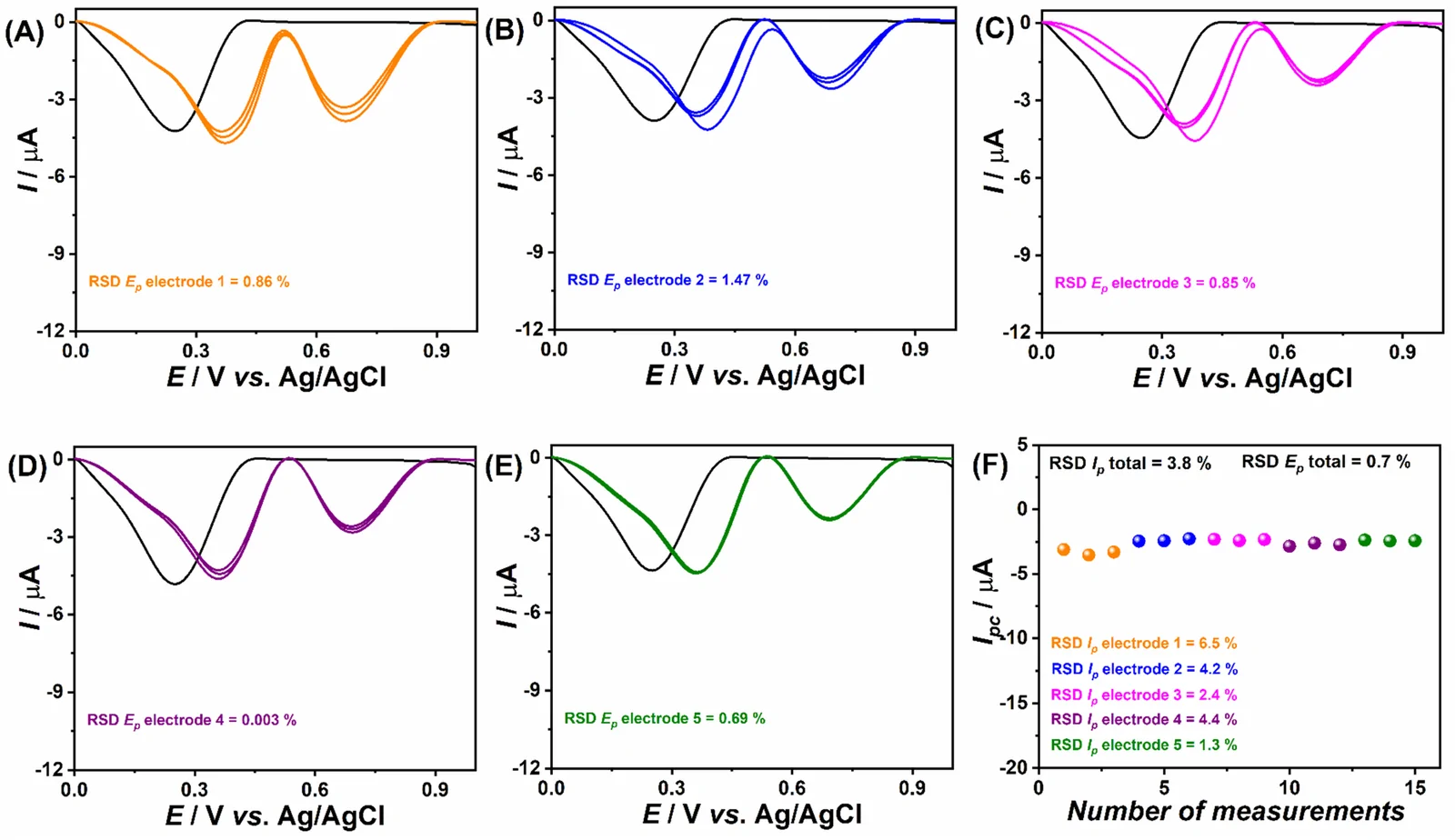
Differential pulse voltammograms in 0.01 mol L^−1^ ammonium chloride solution (blank: black line) before and after addition of 0.1 mg mL^−1^ scopolamine on bulk modified BG-SPE 1 (orange) (A), bulk modified BG-SPE 2 (blue) (B), bulk modified BG-SPE 3 (magenta) (C), bulk modified BG-SPE 4 (purple) (D) and bulk modified BG-SPE 5 (olive) (E). (F) Plot of *I*_p_*vs.* number of measurements performed on five different SPEs-BG. Experimental conditions: amplitude of 90 mV, step potential 10 mV, 50 ms modulation time and 0.2 s time interval; all scans performed in the cathodic direction.

The proposed method demonstrates consistent measurements across various assays using bulk modified BG-SPEs (Fig. 4). The electrochemical responses for scopolamine exhibited good stability, with low RSD_total_ for *E*_p_ (<0.7%) and *I*_p_ (<3.8%). These findings highlight the stability and reproducibility of the laboratory-made screen-printed electrode modified with bromocresol green. Consequently, the bulk modified BG-SPEs, combined with differential pulse voltammetry proves to be an effective and reliable screening method for scopolamine detection.

Next, the linear working range for scopolamine determination on bulk modified BG-SPEs was evaluated using standard solutions of the analyte ranging from 5.0 to 100.0 µg mL^−1^, as shown in Fig. 5A. A linear range (*R*^2^ > 0.992) from 5.0 to 80.0 µg mL^−1^ was established for the quantification of scopolamine, with the linear regression equation: *I*_p_ (µA) = −0.19 (±0.03) − 20.3 (±0.7) × [SCP] (µg mL^−1^). The theoretical/calculated limit of detection (LOD) was estimated as 0.02 µg mL^−1^ using the equation 3*S*_B_/*m*, where *S*_B_ represents the standard deviation (*N* = 10) of the background (blank) signal, and *m* is the slope of the calibration curve. Similarly, the limit of quantification (LOQ) was determined to be 0.07 µg mL^−1^ using the equation 10*S*_B_/*m*.

**Fig. 5:**
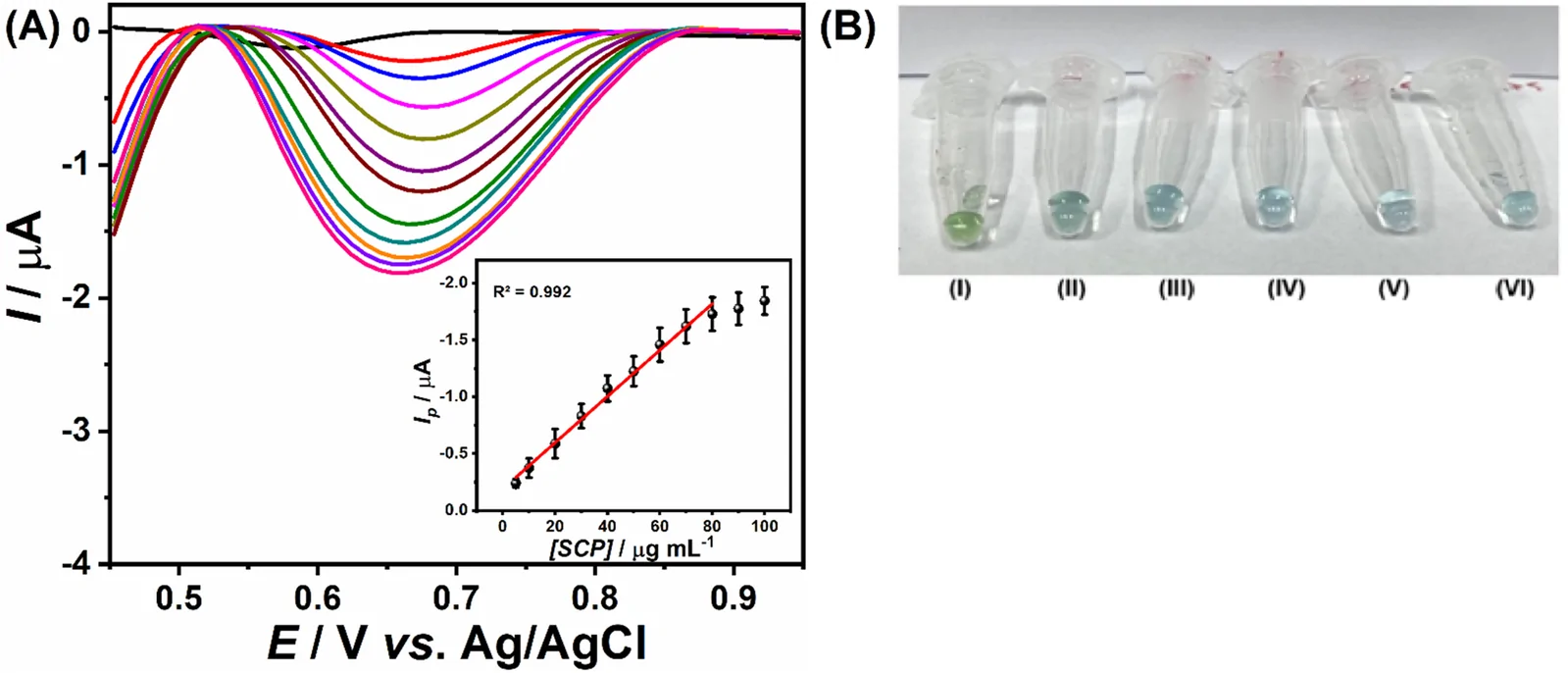
(A) Differential pulse voltammograms in 0.01 mol L^−1^ ammonium chloride solution (blank: black line) on bulk modified BG-SPE before and after addition of 5.0–100.0 µg mL^−1^ scopolamine [SCP]. The experimental conditions were the same as in Fig. 4. Linear regression inserted. All measurements were performed in triplicate and error bars are shown, scans performed in the cathodic direction. (B) Results of the colorimetric step for scopolamine solutions in concentrations from 30 to 303 µg mL^−1^.

The experimentally determined minimum detectable and quantifiable concentrations were both 5.0 µgmL^−1^. It is important to note that while the proposed method can identify and quantify scopolamine over a broad concentration range, this molecule poses a risk to human health only at doses exceeding 20 mg kg^−1^.^32^ A distinct colorimetric response was observed for scopolamine concentrations as low as 30.3 µg mL^−1^ (Fig. 5B), corresponding to the lowest concentration at which the green-to-blue transition could be visually distinguished from the blank (green). The difference between the colorimetric minimum visually distinguishable concentration and electrochemical LODs was expected, given that the colorimetric method relies on direct visual discrimination without image acquisition software or instrumental signal processing.

The colour change observed upon applying the scopolamine solution onto the electrode (prior to electrochemical measurement) makes the method particularly attractive for rapid on-site screening; electrochemical measurement can then be performed on the same platform to provide analytical confirmation and quantitative information. Consequently, the colorimetric response is intended for rapid preliminary screening to qualitatively determine the presence of scopolamine, whereas quantitative determination is based on electrochemical measurement.

Substances commonly found in drinks or biological samples that could potentially interfere with the quantification of scopolamine were evaluated using the proposed method, and the results are presented together with those for scopolamine in Fig. 6A and S5A. Although the colorimetric response alone produced false positive responses for some analytes (Fig. 6A), such as glucose and creatine, which also produced a blue coloration upon contact with the bulk modified BG-SPE surface. The colour change observed with glucose and creatine are likely related to the weakly basic character of these substances under the conditions studied. Given that the colour changes of BG results from a pH-driven acid-base response (Scheme S1), the fact that glucose and creatinine produce the same false-positive response highlights a limitation in the specificity of the colorimetric detection using BG. The electrochemical signal provided a selective response for screening scopolamine against the evaluated interferents, illustrating the complementary nature of the proposed dual-response sensing strategy.

**Fig. 6:**
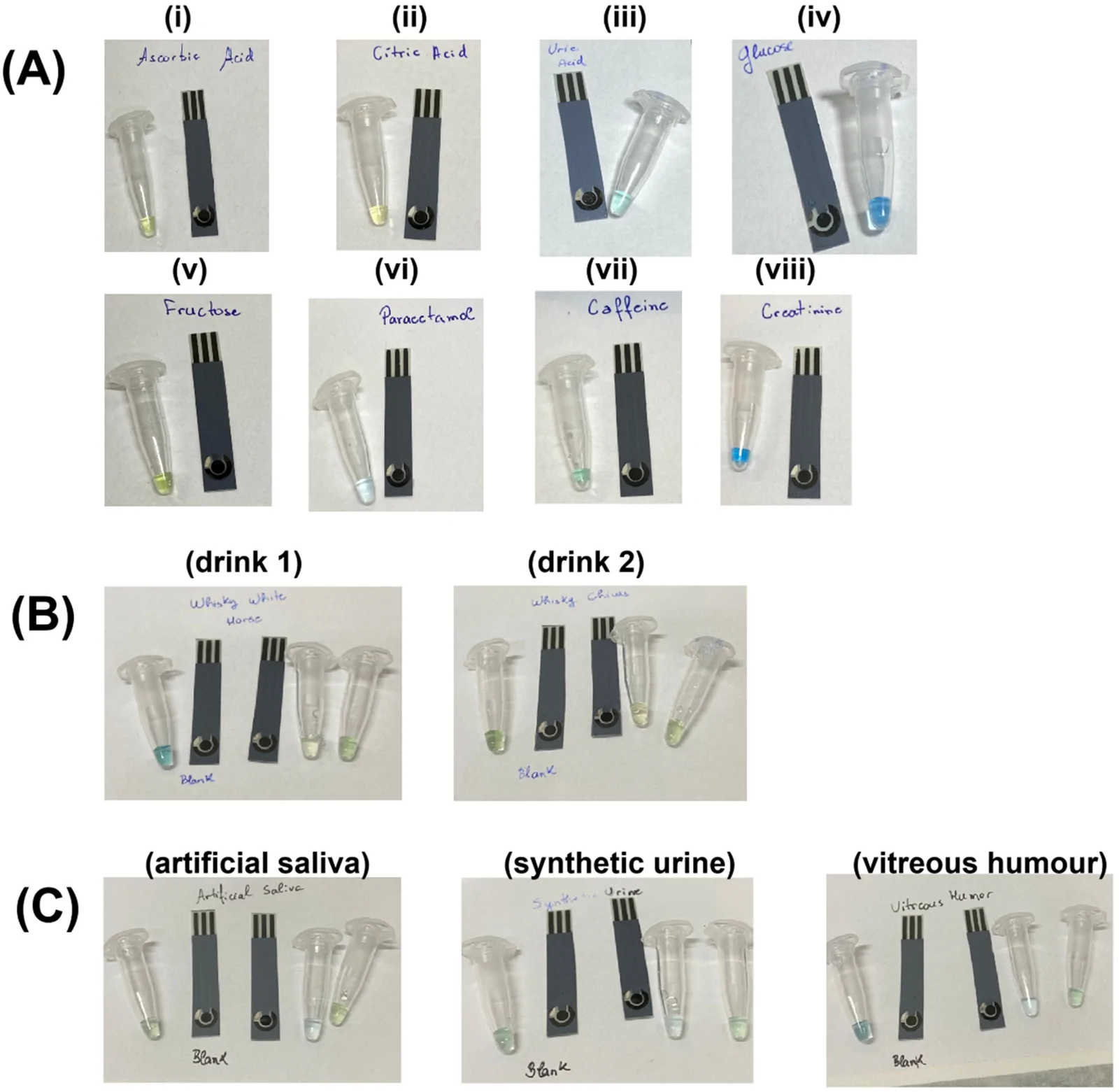
Results for colorimetric step on bulk modified BG-SPEs of interferents at 0.05 mg mL^−1^ (A): AA (i), CA (ii), UA (iii), GLU (iv), FRU (v), PAR (vi), CAF (vii) and creatine (viii). Samples of drinks (B), artificial saliva, synthetic urine and artificial vitreous humour (C) before (blank – the left) and after the addition of 0.05 and 0.1 mg mL^−1^ scopolamine. All compounds in 0.01 mol L^−1^ ammonium chloride solution on bulk modified BG-SPEs.

Fig. S5A shows that AA, CA, UA, GLU, FRU, CAF, and creatine did not exhibit any redox process under the studied conditions. Among the substances tested, only paracetamol (PAR – wine line) showed a reduction peak at +0.45 V (*vs.* Ag/AgCl), which is sufficiently separated from the reduction potential of scopolamine (+0.7 V *vs.* Ag/AgCl), enabling selective detection without signal overlap. Furthermore, beverage samples (whiskeys), artificial saliva, synthetic urine, and artificial vitreous humour were spiked with scopolamine and successfully analysed using the proposed method (Fig. S5B–F and 6).

Certain complex matrices, such as whiskey, interfered with the colorimetric detection of scopolamine at low concentrations, as shown in Fig. 6. This behavior is inherent to pH-indicator-based colorimetric system. The behavior observed is attributed to the intrinsic pH of these samples, which also produces a response from BG on the electrode surface. This completely masks the response to scopolamine, as the colorimetric step responds to pH changes that are correlated with, but not specific to, scopolamine. Such interference was expected, since the colorimetric reagent used for electrode modification is a pH indicator (BG). This behavior also explains why the reduction process of bromocresol green itself exhibited distinct behavior across different samples, indicating that the *I*_p_ and *E*_p_ responses for this redox process are not fully reliable. The potential shift (*E*_pBG_) remained consistently observed only in the presence of scopolamine, providing an additional qualitative indicator of the presence of this compound. These observations highlight the rationale for integrating colorimetric and electrochemical responses into a single detection platform: while the colorimetric response provides immediate visual screening, the electrochemical response offers the selectivity needed to distinguish scopolamine from interferents and complex sample matrices, while using the same solution droplet and sensing interface.

In addition, Fig. S5B–F demonstrate that the electrochemical signal confers greater selectivity to the proposed method, since the electrochemical behavior of scopolamine in beverages and biological samples remained consistent with that of the standard solution. Thus, the proposed method was shown to be effective for the identification of scopolamine in all five samples evaluated, with satisfactory recovery values ranging from 92.3 (±2.3) to 113.0 (±5.8)% based on the electrochemical step. The recoveries obtained in beverages and biological samples confirm that the electrochemical response remains reliable even when the colorimetric response is influenced by the sample composition. Furthermore, modification of the SPE with BG enables an indirect sensing strategy, as the BG redox peak behavior changes only when it reacts with the analyte (*E*_pBG_ shift), such as scopolamine. This specific interaction was not observed for any of the interferents evaluated (Fig. S5A), further increasing the selectivity of the electrode. This characteristic not only makes the proposed electrode highly suitable for forensic applications but also suggests its potential for detecting other compounds that react with BG, thereby extending its applicability to a wider range of sample types. Future work will be directed to the expanding the range of the real samples. In summary, the results demonstrate that incorporating bromocresol green directly into the SPE ink enables complementary colorimetric and electrochemical responses to be generated from the same sensing interface. While the colorimetric response provides rapid preliminary screening information, the electrochemical response offers the selectivity confirmation and quantitative capability required for reliable determination of scopolamine in complex matrices, while requiring a single sensing platform and low sample volumes, using the same droplet.

### Comparison of the electroanalytical methods for scopolamine detection

Table 1 presents a comparison of the main analytical parameters for scopolamine determination using electroanalytical methods reported to date.

**Table 1:** Electroanalytical methods for scopolamine determination^a^

Technique	Working electrode	Linear range (µg mL^−1^)	LOD (µg mL^−1^)	Sample	Ref.
SWV	BDD	0.3–33.37	0.26	Pharmaceutical formulations	7
DPV	CPE-HMS-MPY	0.273–60.67	0.091	Tea	9
BIA-SWV	BDD	0.3–4.25	0.042	Drinks	8
DPV	Pt	0.49–303.35	0.06	Pharmaceutical formulations	10
SWV	SPGE	24.9–225.1 and 24.9–175.0	5.01 and 8.01	Drinks and biological samples	17
DPV	Bulk modified BG-SPE	5.0–80.0	0.02	Drinks and biological samples	This work

a SWV: square-wave voltammetry; DPV: differential pulse voltammetry; BIA: batch injection analysis; BDD: boron-doped diamond; CPE-HMS-MPY: carbon paste electrodes modified with mesostructured silicas; Pt: platinum.

Although the proposed method demonstrates competitive analytical performance, including a favourable linear range, low LOD, and minimum experimentally detectable concentration (5.0 µg mL^−1^) compared with other reported electroanalytical approaches (Table 1), the principal innovation lies in the integrated dual-sensing platform, which requires only a minimal sample volume (45 µL) and eliminates the requirement for complex electrochemical cell assemblies.

The lower calculated LOD (0.02 µg mL^−1^) obtained for the BG-SPE compared with those reported in previous studies (Table 1) should not be interpreted as direct evidence of an electrocatalytic effect arising from BG incorporation. Instead, this comparatively low LOD is more likely related to the specific DPV measurement conditions and the resulting analytical sensitivity than to an intrinsic electrocatalytic effect of the BG modification. Importantly, despite the low calculated LOD, a reliable linear response was experimentally obtained only from a scopolamine concentration of 5.0 µg mL^−1^ onwards.

The proposed method for scopolamine detection in forensic samples involves a straightforward sample preparation procedure: methanol extraction followed by dilution in the supporting electrolyte. This approach offers clear advantages for forensic applications. The proposed sample preparation protocol is compatible with both preliminary screening and confirmatory techniques, including LC-MS and GC-MS. Moreover, this strategy reduces costs and sample consumption, enhances productivity, and is well-suited for on-site testing.

To our knowledge, this is the first report of a colorimetric reagent-modified electrode that enables complementary colorimetric and electrochemical detection, affording both colorimetric and electrochemical analytical responses. Unlike previously reported works, the present platform integrates a colorimetric reagent directly into the screen-printing ink, enabling two complementary analytical responses to be obtained from the same sensing interface without additional derivatization or reagent addition. Furthermore, this work includes an extensive interference study that demonstrates the robustness of the method for selective identification of scopolamine. Even when the colorimetric step yields false-positive or false-negative results, the electrochemical step ensures the selectivity required for real-world applications; these features distinguish the proposed sensor from previously described platforms (including those with dual detection capabilities) by integrating colorimetric and electrochemical functionalities into a single disposable sensing platform. This design enables immediate visual screening followed by selective electrochemical confirmation using the same sample droplet, without the addition of external reagents or transfer of the sample to a second analytical platform.

## Conclusion

This study demonstrates, for the first time, the incorporation of bromocresol green directly into graphite ink to fabrication a bulk-modified SPE capable of generating complementary colorimetric and electrochemical responses from the same sensing interface. This configuration enables two distinct analytical responses to be generated within the same sensing interface: a colour change associated with the reaction of BG and a characteristic electrochemical signal. The incorporated BG enabled the sample droplet to change colour from green (in the absence of scopolamine) to blue in its presence. The colorimetric step provides a pH-based response correlated with, but not specific to, scopolamine, whereas greater specificity is provided by the electrochemical step. The technique was employed to establish a portable dual-response screening strategy for scopolamine detection, achieving an LOD of 0.02 µg mL^−1^ for the electrochemical step, with the minimum visually distinguishable concentration from 30 µg mL^−1^. The colorimetric response enables rapid preliminary screening, whereas the electrochemical response provides selective confirmation and quantitative determination. The proposed platform provides a working electrode capable of providing a sensitive, low-cost, and rapid colorimetric and electrochemical response for use as a scopolamine screening method without requiring external reagent addition, making it a useful tool for preliminary forensic screening with electrochemical confirmation using the same sensing platform. The proposed strategy may also be extended to other colorimetric reagents, providing a versatile route for the development of integrated dual-response sensors.

## Author contributions

The manuscript was written with the contribution of all authors. The final version has been approved by all authors.

## Conflicts of interest

The authors declare that they have no known competing financial interests or personal relationships that could have appeared to influence the work reported in this paper.

## Supplementary Material

RA-OLF-D6RA07984A-s001

## Data Availability

The data supporting this article have been included as part of the supplementary information (SI). Supplementary information is available. See DOI: https://doi.org/10.1039/d6ra07984a.
