# Supplementary material for: Forensic electrochemistry on a drop: bromocresol green-modified screen-printed platforms for dual colorimetric-electrochemical detection of scopolamine
Source: RSC Adv. 2026 Oct 6;16(46):60505–15. doi: 10.1039/d6ra07984a (PMC13640614; doi:10.1039/d6ra07984a)
Supplement: Supplementary file 1 [file RA-016-D6RA07984A-s001.pdf]

Supplementary Information (SI) for RSC Advances.
This journal is © The Royal Society of Chemistry

Supporting Information:

Forensic Electrochemistry on a Drop: Bromocresol Green-Modified Screen-Printed Platforms for Dual Colorimetric-Electrochemical Detection of Scopolamine

Larissa M. A. Melo[a,b], Robert D. Crapnell[a], Elena Bernalte[a], Daniel S. Paula[c], Rodrigo A. A. Munoz[d], Wendell K. T. Coltro[c], Wallans T. P. dos Santos[e*] and Craig E. Banks[a*]

[a] *Faculty of Science and Engineering, Manchester Metropolitan University, Dalton Building, Chester Street, M1 5GD, Great Britain.*

[b] *Department of Chemistry, Federal University of Vales do Jequitinhonha and Mucuri, Campus JK, 39100000, Diamantina, Minas Gerais, Brazil*

[c] *Department of Chemistry, Federal University of Goiás, Campus Samambaia, 74690900, Goiânia, Goiás, Brazil*

[d] *Institute of Chemistry, Federal University of Uberlândia, 38400-902, Uberlândia, Minas Gerais, Brazil*

[e] *Department of Pharmacy, Federal University of Vales do Jequitinhonha and Mucuri, Campus JK, 39100000, Diamantina, Minas Gerais, Brazil*

*To whom correspondence should be addressed.

E-mail: wallanst@ufvjm.edu.br and c.banks@mmu.ac.uk; Tel: +44(0)1612471196

Surface characterization

Surface morphologies of the different bulk modified BG-SPE prepared were examined using a Crossbeam 350 Focussed Ion Beam – Scanning Electron Microscope (FIB-SEM) (Carl Zeiss Ltd., Cambridge, UK) fitted with a field emission electron gun. Secondary electron imaging was completed using a Secondary Electron Secondary Ion (SESI) detector. Energy Dispersive X-ray Spectroscopy (EDX) analysis was performed using an Ultim Max 170 detector (Oxford Instruments plc, Abingdon, UK.) installed on the FIB-SEM. An acceleration voltage of 20 kV was used to image samples for EDX analysis experiments. Samples were mounted on aluminium SEM pin stubs (12 mm diameter, Agar Scientific, Essex, UK) using adhesive carbon tabs (12 mm diameter, Agar Scientific, Essex, UK) and coated with a 5 nm Au/Pd layer using a Leica EM ACE200 coating system before imaging. Quantification of EDX spectra was performed using a standardless quantification procedure developed by Oxford Instruments and integrated into AZtec 6.1 (Oxford Instruments, Abingdon, UK).

X-ray photoelectron spectroscopy (XPS) data using an AXIS Supra (Kratos, UK), equipped with a monochromatic Al X-ray source (1486.6 eV) operating at 225 W and a hemispherical sector analyzer were used for characterization. It was operated in fixed transmission mode with a pass energy of 160 eV for survey scans and 20 eV for region scans with the collimator operating in slot mode for an analysis area of approximately 700 × 300 μm, the FWHM of the Ag 3d5/2 peak using a pass energy of 20 eV was 0.613 eV. The binding energy scale was calibrated by setting the graphitic sp2 C 1s peak to 284.5 eV. This calibration is recognized as

imperfect, [1] yet it was employed due to the lack of viable alternatives and because only limited conclusions could be drawn from the absolute peak positions. Raman spectroscopy was performed on a DXR Raman Microscope (Thermo Scientific Inc., Waltham, MA, USA) configured with a 532 nm laser and operated using OMNIC 9 software.

Electrochemical measurements

Voltammetric experiments were conducted using a PGSTAT 204 potentiostat (Metrohm Autolab BV, Utrecht, The Netherlands), controlled by NOVA 2.1.5 software. Electrochemical studies were carried out with laboratory-produced bulk modified BG-SPEs to investigate the behaviour of SCP under varying pH conditions and scan rates (ν) using cyclic voltammetry. Before each measurement, the bulk modified BG-SPEs were electrochemically conditioned in a 10 mmol L^{-1} ammonium chloride solution by two successive scans within a potential range of – 0.2 V to + 1.3 V (*vs.* Ag/AgCl) at a ν of 50 mV s^{-1}. Note only the bulk modified BG-SPEs pseudo-reference electrodes (Ag/AgCl) were used throughout, and the analyses were performed in drops. Square wave voltammetry (SWV) and differential pulse voltammetry (DPV) were evaluated and optimized for the detection of SCP. Among these techniques, DPV was selected for its superior performance, using the following optimized parameters: an amplitude of 90 mV, a step potential of – 10 mV, a modulation time of 50 ms, and a time interval of 0.2 s. All measurements were performed in triplicate.

The electroactive areas of the SPEs and bulk modified BG-SPEs were calculated using the *quasi*-reversible Randles–Ševćik equation and cyclic voltammetry. Cyclic voltammetry scan rates of 5, 10, 15, 25, 50, 75, 100, 150, and 250 mV s^{-1} was employed, and the final area values were calculated as the mean of three measurements (n = 3). The limits of detection and quantification were calculated according to IUPAC recommendations. [2] Recovery values were calculated using the peak current responses of the samples and the linear regression equation obtained from the calibration curve (n = 3).

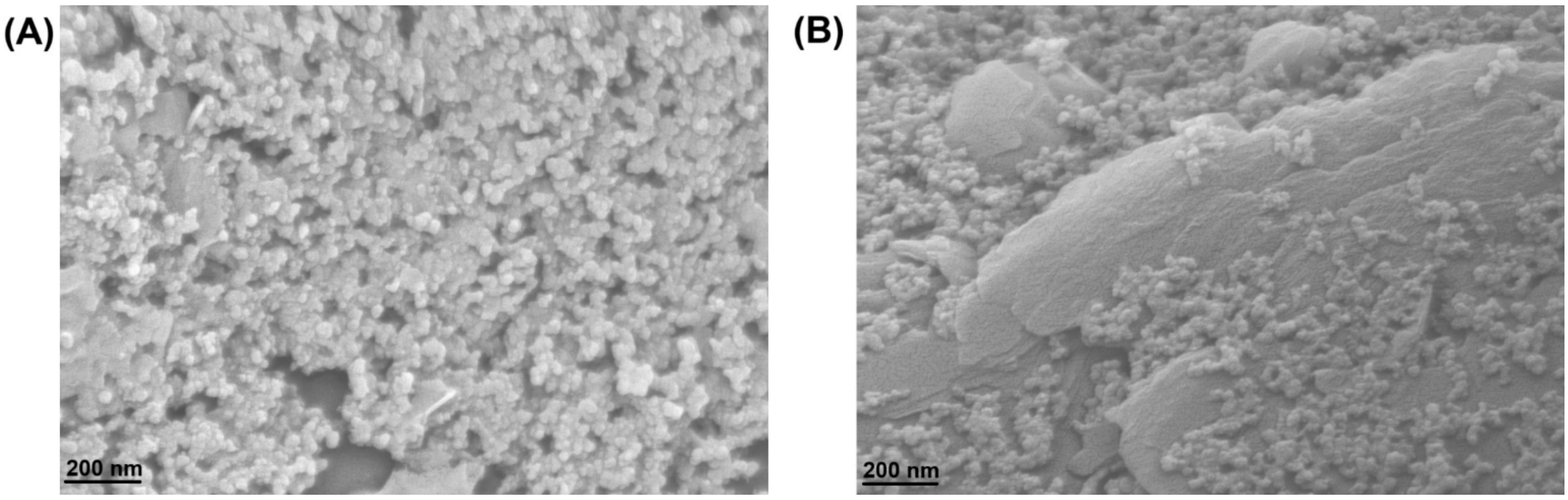

Figure S1. SEM imagines the SPE surface before (**A**) and after (**B**) modification with BG at 50.00 kx magnification.

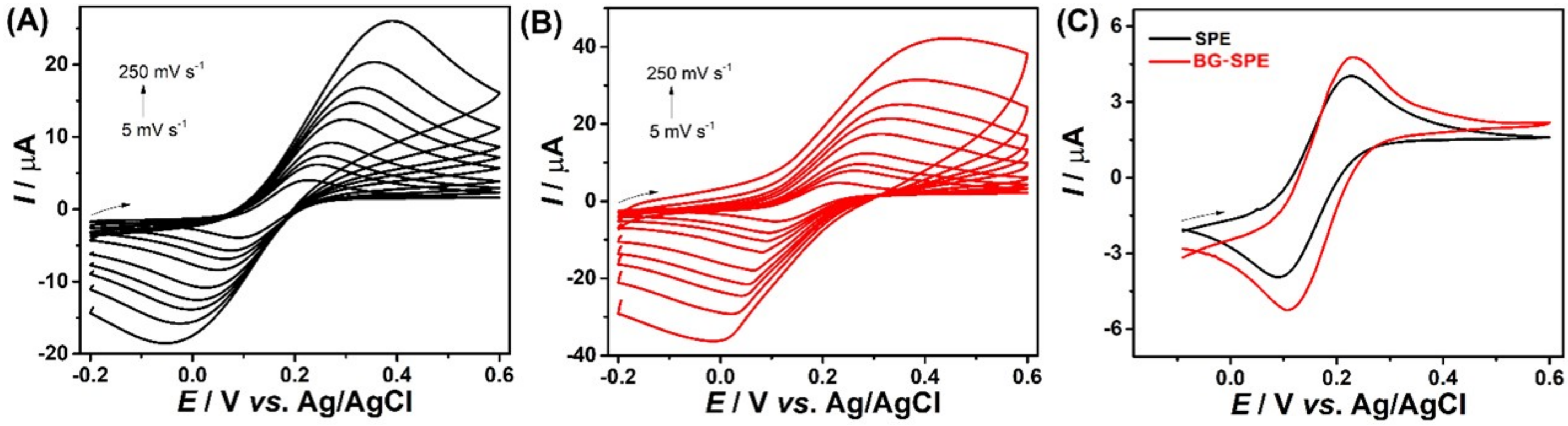

Figure S2. Cyclic voltammograms for 1.0 mmol L^{-1} $[Fe(CN)_6]^{4-}$ / 0.1 mol L^{-1} KCl recorded at scan rates from 5 to 250 mV s^{-1} on SPE (**A**) and bulk modified BG-SPEs (**B**), and comparison at 5 mV s^{-1} on SPE and bulk modified BG-SPEs (**C**).

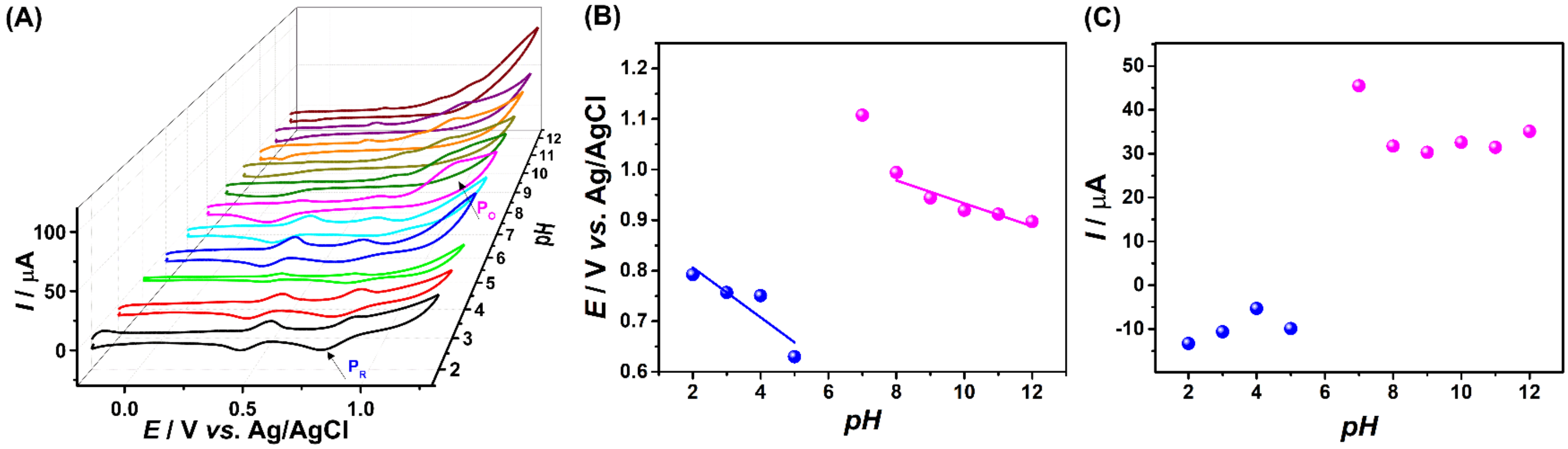

Figure S3. (**A**) 3D plots of recorded cyclic voltammograms (first scan) of 0.1 mg mL⁻¹ scopolamine in 0.1 mol L⁻¹ BR buffer solution with different pH values (from 2.0 to 12.0). All potential scans started at − 0.2 V (*vs.* Ag/AgCl), with a scan rate of 25 mV s⁻¹. Plot of (**B**) E_p and (**C**) I_p *vs.* pH for the scopolamine redox processes on bulk modified BG-SPEs. Oxidation in magenta and reduction in blue.

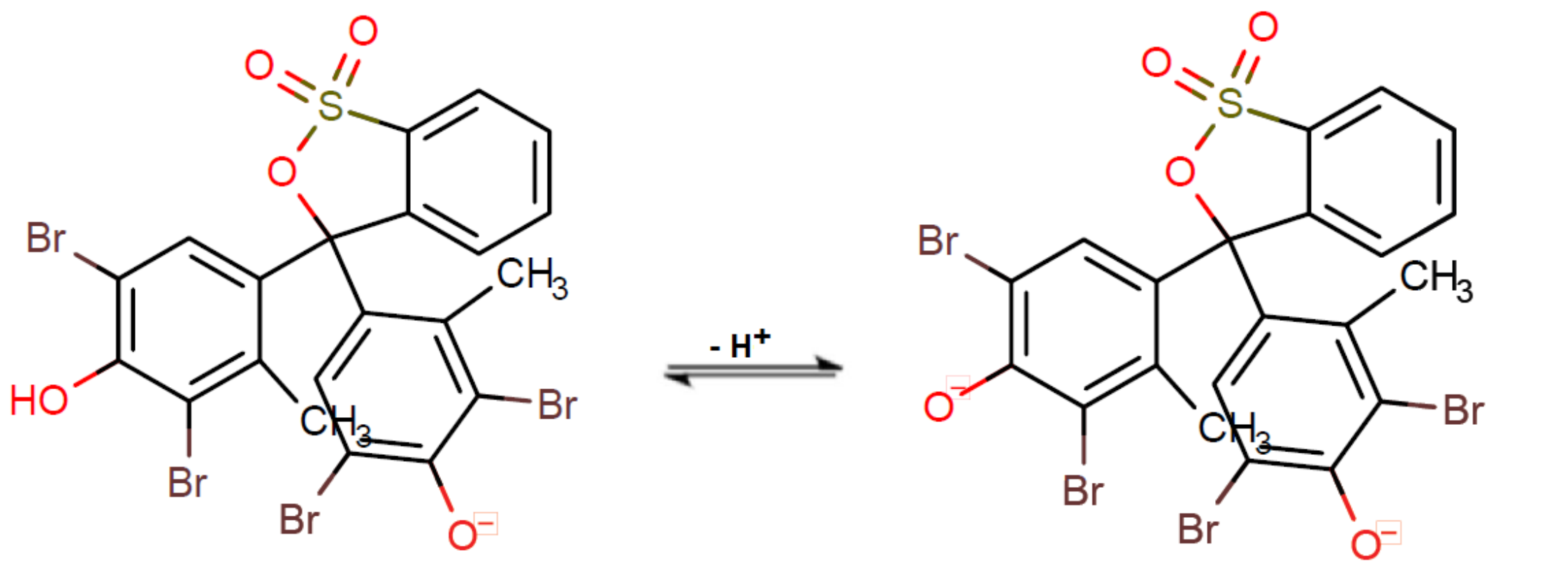

Scheme S1. Mechanism for the colour change of BG from green to blue in response to a change in the medium's pH.

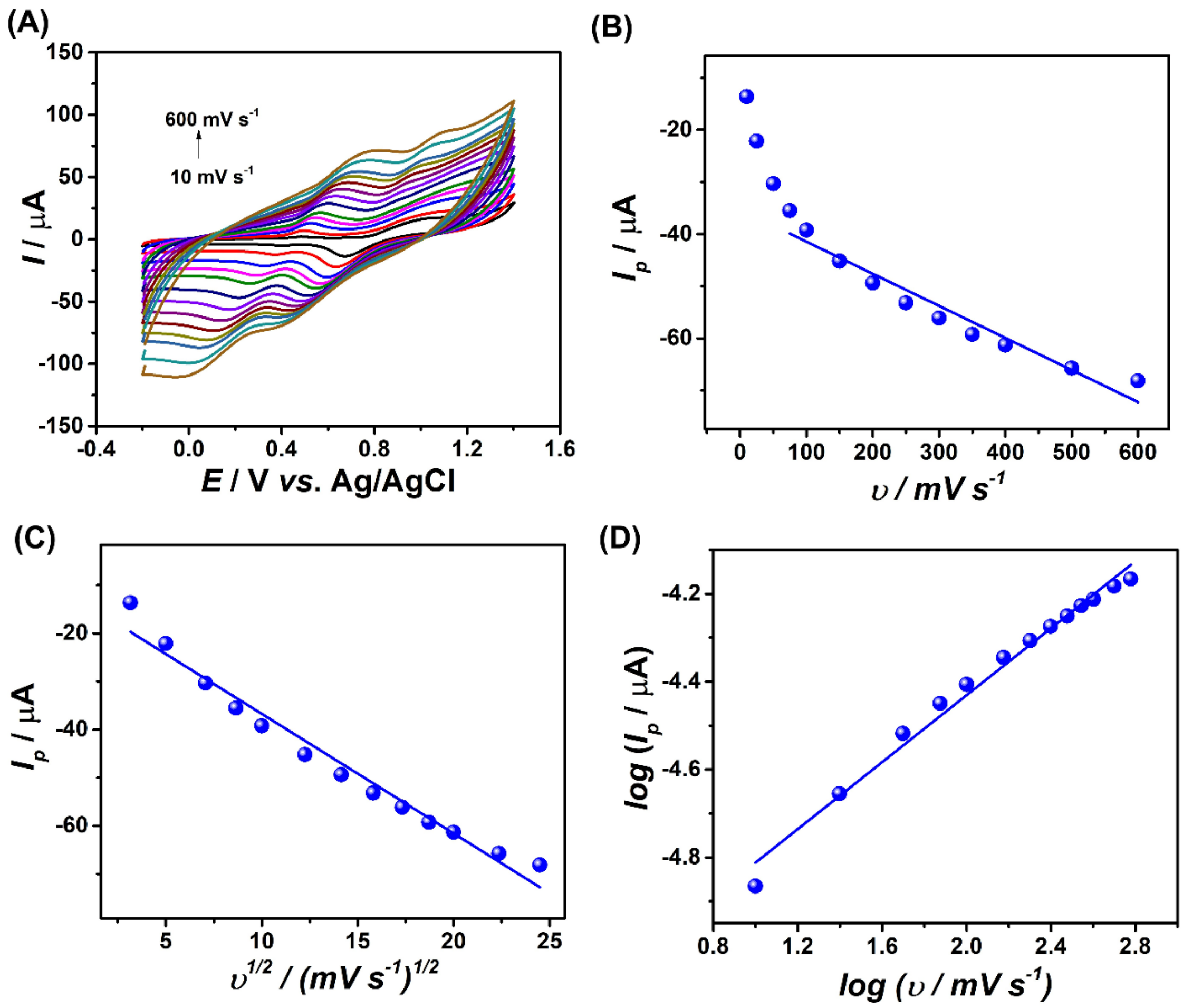

Figure S4. (**A**) Cyclic voltammograms recorded at scan rates from 10 to 600 mV s⁻¹ on bulk modified BG-SPEs for 0.1 mg mL⁻¹ scopolamine in 0.01 mol L⁻¹ ammonium chloride solution. Plots of the linear regressions obtained for (**B**) I_p *vs.* v; (**C**) I_p *vs.* $v^{1/2}$, and (**D**) log I_p *vs.* log v.

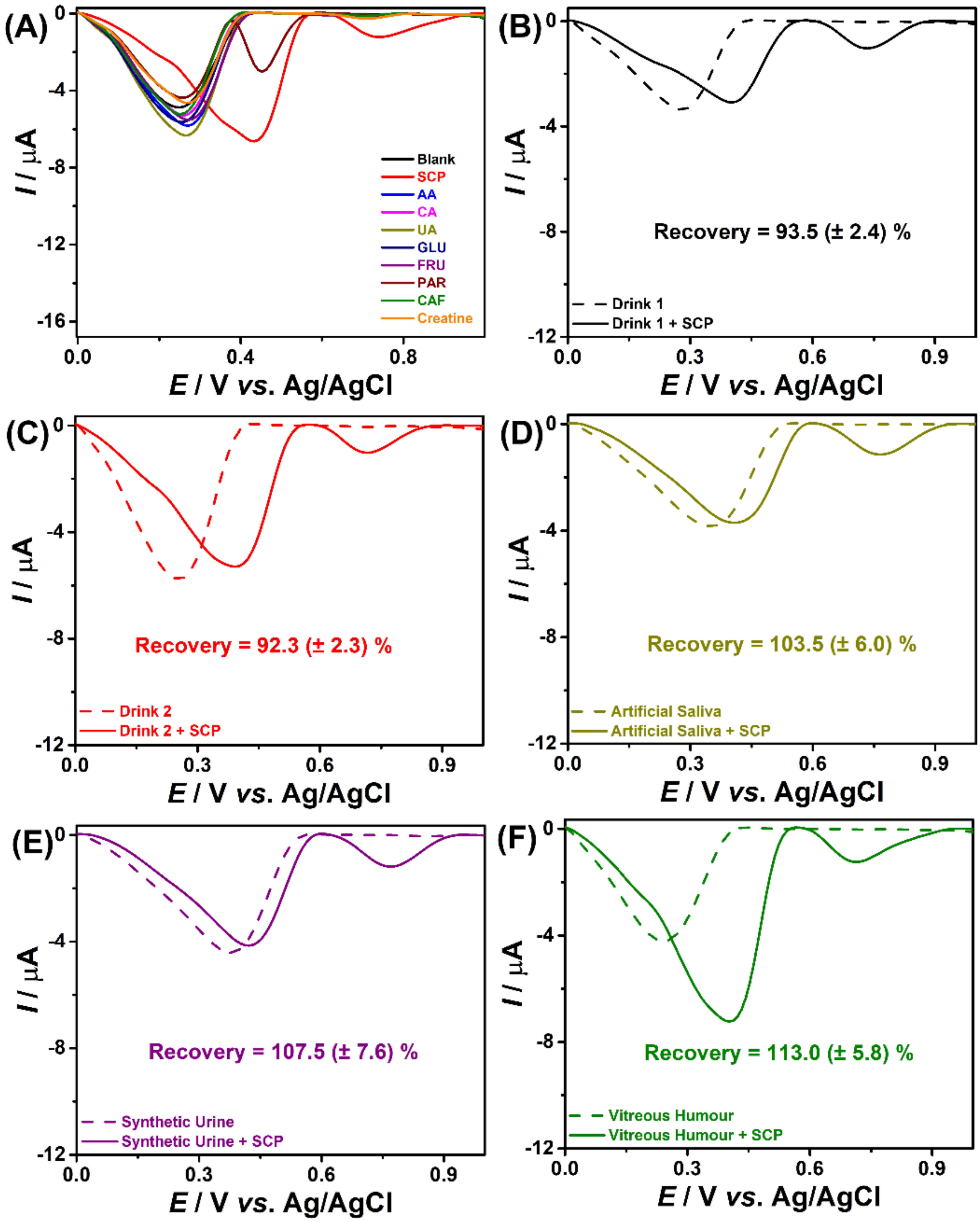

Figure S5. (**A**) Differential pulse voltammograms of 50 μg mL^{-1}: scopolamine (SCP), AA, CA, UA, GLU, FRU, PAR, CAF and creatine. Samples of drinks (**B** and **C**), artificial saliva (**D**), synthetic urine (**E**) and artificial vitreous humor (**F**), in dashed lines before and in solid lines after addition of 50 μg mL^{-1} scopolamine. All compounds in 10 mmol L^{-1} ammonium chloride solution. Recoveries inserted. The experimental conditions were the same as in Fig. 4.

References

1. G. Greczynski and L. Hultman, *Scientific Reports*, 2021, **11**, 11195.
2. J. Mocak, A. M. Bond, S. Mitchell and G. Scollary, *Pure and Applied Chemistry*, 1997, **69**, 297-328.
